# The Fabrication and Characterization of Surface-Acoustic-Wave and Resistive Types of Ozone Sensors Based on Zinc Oxide: A Comparative Study

**DOI:** 10.3390/s25092723

**Published:** 2025-04-25

**Authors:** Sheng-Hua Yan, Chia-Yen Lee

**Affiliations:** Department of Materials Engineering, National Pingtung University of Science and Technology, Pingtung 912, Taiwan; m11345004@st.npust.edu.tw

**Keywords:** MEMS, ozone sensing, resistive sensor, surface acoustic wave, zinc oxide

## Abstract

Micro-Electro-Mechanical System (MEMS) technology is employed to fabricate surface acoustic wave (SAW)-type and resistive-type ozone sensors on quartz glass (SiO_2_) substrates. The fabrication process commences by using a photolithography technique to define interdigitated electrodes (IDEs) on the substrates. Electron-beam evaporation (EBE) followed by radio frequency (RF) magnetron sputtering is then used to deposit platinum (Pt) and chromium (Cr) electrode layers as well as a zinc oxide (ZnO) sensing layer, respectively. Finally, annealing is performed to improve the crystallinity and sensing performance of the ZnO films. The experimental results reveal that the ZnO thin films provide an excellent ozone-concentration sensing capability in both sensors. The SAW-type sensor demonstrates a peak sensitivity at a frequency of 200 kHz, with a rapid response time of just 35 s. Thus, it is suitable for applications requiring a quick response and high sensitivity, such as real-time monitoring and high-precision environmental detection. The resistive-type sensor shows optimal sensitivity at a relatively low operating temperature of 180 °C, but has a longer response time of approximately 103 s. Therefore, it is better suited for low-cost and large-scale applications such as industrial-gas-concentration monitoring.

## 1. Introduction

Toxic and flammable gases play an invaluable role in many industrial and everyday applications, including carbon monoxide in metal fabrication, hydrogen sulfide in analytical chemistry, ammonia in refrigeration systems, chlorine in water treatment, ethanol in pharmaceuticals and beverages, and ozone in air purification. The widespread use of these gases poses serious health and safety concerns, indicating the need for effective toxic-gas monitoring solutions.

Ozone, a common toxic gas, is widely used in industry and has many applications in daily life. However, excessive exposure to ozone can cause severe health issues, including asthma, lung-function impairment, alveolar damage, and even death. Studies have shown that even a slight increase in the ozone concentration can have significant adverse effects. For example, an average daily ozone-concentration increase of 50 ppb can result in a decline in lung function of more than 4% in patients with asthma. Therefore, the real-time monitoring of ozone concentration is essential to protect human health [1].

Doll et al. designed an ozone sensor based on conductivity variations utilizing a pure In_2_O_3_ thin film as the sensing material. The sensor exhibited stable conductivity variation slopes at operating temperatures of 40 and 130 °C, indicating excellent electronic transport stability under these conditions. Moreover, the sensor showed a response time of approximately 2.5 h at 130 °C [2]. Berry et al. conducted an in-depth study on the electrical response of tin dioxide (SnO_2_) ozone sensors under atmospheric conditions. The sensor utilized a thermal insulation design to isolate the sensing area from the silicon substrate, thereby enhancing the energy utilization efficiency and minimizing the impact of external thermal disturbances [1].

Micro-Electro-Mechanical System (MEMS) technology is a multidisciplinary field encompassing mechanics, electronics, and materials science [3,4,5]. Bendahan et al. fabricated an ozone sensor composed of a WO_3_-based sensing layer using a MEMS, Pt electrodes, and SiO_2_/Si substrates [6]. Owing to the inherently high resistance of WO_3_, the Pt electrode was designed with an interdigitated structure to shorten the current path and increase the conductivity. Experiments were conducted to compare the performance of sensors fabricated with different oxygen-to-argon ratios under an environment of 800 ppb of ozone. The impact of the working temperature on the sensitivity of the sensor was also investigated. The sensor sputtered with an oxygen-to-argon ratio of 1:1 showed the lowest conductance (and hence the best detection performance) among the considered sensors. Furthermore, the sensitivity and stability of the sensor were enhanced at a working temperature of 523 K.

Gases are used in many industrial applications, including preservation, packaging, refining, etching, and cryogenic freezing. Industrial gases, such as chlorine (Cl_2_), nitrogen dioxide (NO_2_), and hydrogen sulfide (H_2_), are often harmful to human health and overall well-being. Therefore, the development of effective gas sensors is of the utmost importance in ensuring worker safety, environmental protection, and regulatory compliance. Many different types of gas sensors are available, where each sensor generally leverages a strong linear relationship between the gas concentration and the output signal [7,8,9,10,11,12,13,14,15,16,17,18].

In the study of da Silva et al. [12], they developed ozone sensors based on ZnO-SnO_2_ heterojunctions that were prepared using a hydrothermal route. Their working temperatures were reduced to room temperature under continuous UV enhancement, even as low as 20 ppb. Furthermore, de Lima et al. [13] investigated reduced graphene oxide (rGO)-ZnO nanocomposites on ozone-gas-sensing properties. A reduction process of GO was carried out using UV laser radiation followed by a sputtering deposition of ZnO nanoparticles on the rGO surface. The developed material could be operated at high temperatures and exhibited a limit of detection of 100 ppt. Nagarjuna et al. [15] fabricated an ozone-gas sensor using a high-power impulse magnetron sputtering (HiPIMS) process with nanoporous ZnO as the sensing material. The sensor showed a high sensing response at a 200 °C operating temperature and with a 400 °C annealing temperature. The best sensing response characteristics ranged from 50 ppb to 1000 ppb, but the sensing response decreased as the relative humidity increased due to the moisture adsorption and desorption phenomenon on the surface of the ZnO sensor. Komorizono et al. [17] synthesized a porous ZnO nanostructure using a microwave-assisted hydrothermal method, followed by calcination to induce pore formation. The porous ZnO sensor demonstrated the ability to detect low ozone concentrations, e.g., 50 ppb, and exhibited quick response and recovery times with a high selectivity to NO_2_, NH_3_, and CO_2_.

Tang et al. [18] fabricated ozone sensors consisting of ZnO thin films deposited by radio-frequency magnetron sputtering, which were then annealed at temperatures ranging from 300 to 500 °C for 3 to 5 h in order to promote surface crystallization. Among the various sensors, the sensor that annealed at 500 °C showed the best sensing performance for all values of the working temperature. For ozone concentrations ranging from 1 to 5 ppm, the optimal response was obtained at a working temperature of 190 °C. Moreover, under the maximum ozone concentration of 5 ppm, the response was reached in as quickly as 100 s. Overall, the results indicated the importance of the annealing temperature and operating conditions in enhancing the sensitivity and stability of the ZnO-based ozone sensor for applications in environmental monitoring, air quality control, and public health safety.

Surface acoustic wave (SAW) sensors are revolutionary devices spanning the biomedical, chemical, and environmental sensing domains. They have extensive applications in the mechanical, biological, chemical, gas, and microfluidic fields for detecting a wide range of parameters, including viscosity, conductivity, and temperature. Interdigitated transducers (IDTs) generate acoustic waves that propagate through a piezoelectric substrate, where they couple with an electric field and the stress state on the material surface [19].

This study compares the applications of SAW and MOS technologies for ozone sensing using ZnO thin films. Both technologies utilize the adsorption properties of ZnO thin films to detect ozone concentrations through changes in their physical or electrical properties. However, their operating mechanisms and application scenarios differ. In particular, SAW technology relies on the propagation characteristics of acoustic waves on the surface of piezoelectric materials. When ozone molecules are adsorbed by the ZnO thin films, changes in the mass and mechanical properties of the films alter the frequency or phase of the acoustic waves, enabling detection. In contrast, MOS technology determines the ozone concentration by measuring the resistance changes induced by the chemical reaction between the ozone molecules and the ZnO-thin-film’s surface. To improve the sensing performance, both technologies employ interdigitated electrodes (IDEs) to support the ZnO thin films, thereby effectively enhancing the sensitivity and response speed of the sensor.

## 2. Principles and Designs

Metal oxide semiconductor (MOS)-based gas sensors operate at high temperatures, typically between 200 and 400 °C, to detect gas concentrations by measuring changes in the electrical conductivity caused by gas adsorption on the sensor surface. The sensing principle relies on the interaction of the target gas with surface-adsorbed oxygen ions on the metal oxide semiconductor. For example, in n-type semiconductors such as SnO_2_ or ZnO, conduction electrons are transferred to the oxygen molecules on the oxide surface, which are adsorbed in the form of negatively charged ions (O^−^). These ions reduce the charge carrier concentration on the oxide surface. However, when a reducing gas such as carbon monoxide (CO) comes into contact with the sensor, it reacts with the O^−^ ions to form CO_2_ and releases electrons back to the material, thereby reducing the sensor resistance. In p-type semiconductors, where positive holes dominate as charge carriers, oxidizing gases increase the hole concentration, whereas reducing gases decrease it, thereby altering the sensor resistance accordingly. Thus, in MOS-based sensors, the gas concentration can be accurately determined by monitoring the changes in conductivity or resistivity in the presence of the target gas [20].

**Figure 1:**
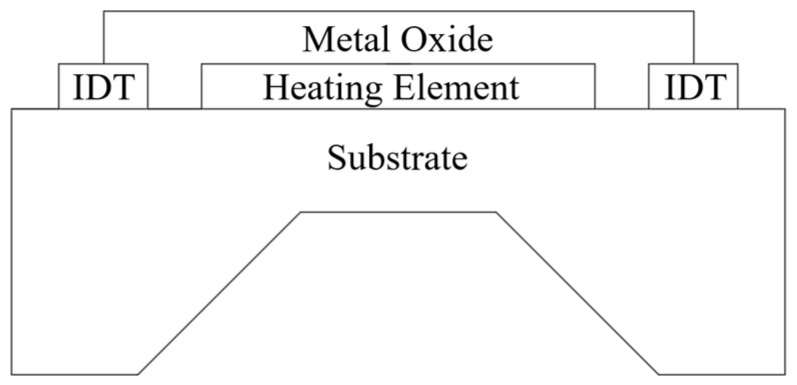
Schematic of metal-oxide-semiconductor gas sensor [21].

As is shown in Figure 1, MOS-based gas sensors typically consist of a metal oxide layer placed between two electrodes on a non-conductive substrate. A built-in heater elevates the substrate temperature to a level where the gas molecules can reversibly modify the conductivity of the semiconductor material, thereby enabling the gas concentration to be reliably measured [21].

Surface acoustic wave (SAW)-type gas sensors utilize the interaction of surface acoustic waves with a sensing film to detect specific gas concentrations. The working principle of SAW-type gas sensors involves generating surface acoustic waves on a piezoelectric substrate using interdigitated transducers (IDTs).

**Figure 2:**
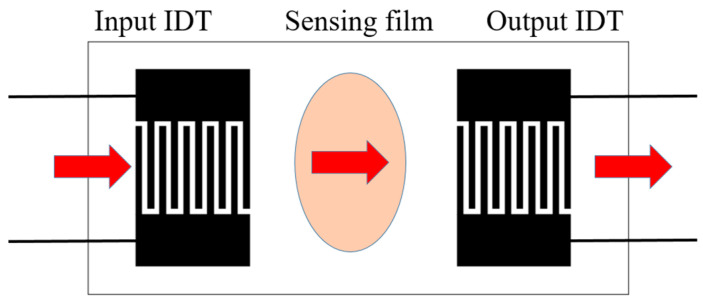
Surface-acoustic-wave gas sensor structure.

SAW-type gas sensors incorporate two IDT electrodes, as shown in Figure 2. When an alternating voltage (V_in_) is applied to the input IDT, it generates surface acoustic waves that propagate along the piezoelectric substrate. The surface of the substrate is coated with a thin sensing film that reacts with the target gas molecules. As the molecules interact with the film, they alter its physical properties, such as its mass or elasticity. These changes modulate the velocity or amplitude of the surface acoustic waves propagating along the substrate. The output IDT detects these variations and converts them into an electrical signal, which is then analyzed to determine the gas concentration [19].

**Figure 3:**
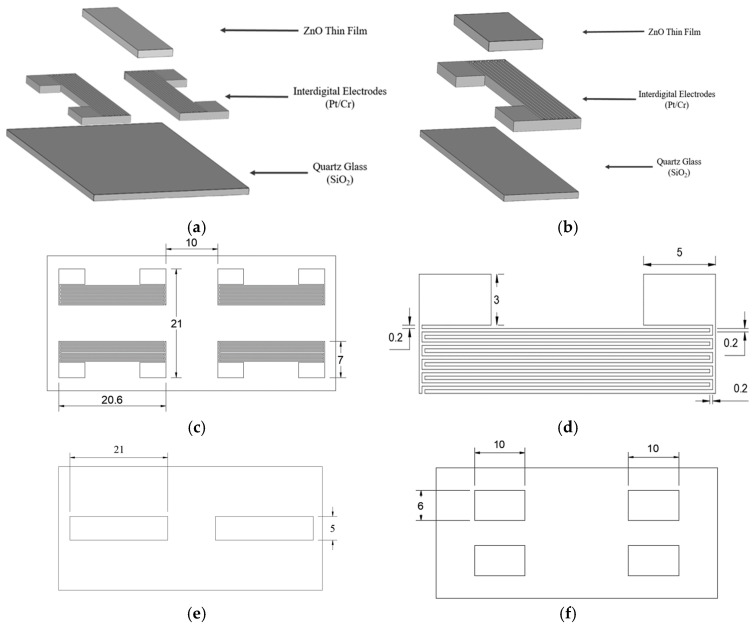
Structural assembly and component details of proposed MEMS-based pyroelectric ozone sensor: (**a**) schematic of SAW-type ozone sensor based on ZnO thin films, (**b**) schematic of resistive ozone sensor based on ZnO thin films, (**c**) dimensions and layout of platinum-electrode-layer mask, (**d**) detailed structure and design of interdigitated electrodes, (**e**) SAW sensing layer, and (**f**) resistive sensing layer (unit: mm).

Figure 3a presents an exploded view of the SAW chip, while Figure 3b shows the main components of the resistive ozone sensor. The ZnO thin films serve as the sensing layer, reacting with the ozone molecules and prompting resistance changes. The interdigitated electrodes, which are made of platinum and chromium, detect the resistance changes while providing a stable electrical performance. Finally, the quartz glass substrate acts as a supporting structure with enhanced acoustic wave transmission and mechanical stability. The overall dimensions of the platinum IDE mask are 21 mm × 20.6 mm, which perfectly matches the size of the quartz glass substrate, as shown in Figure 3c. The mask consists of four symmetrically arranged sets of interdigitated electrodes, with a spacing of 10 mm between them. As is shown in Figure 3d, each electrode bar has a width of 0.2 mm, a distance of 0.2 mm from its neighbors, and a height of 3 mm. The staggered structure of the electrodes ensures an effective field distribution. As is shown in Figure 3e, the mask for the SAW-type sensor features a central sensing region with dimensions of 21 mm × 6 mm, which is designed to facilitate the propagation of the acoustic waves. The mask for the resistive sensor has a smaller size of 10 mm × 6 mm, ensuring a uniform current distribution and stable sensing performance (Figure 3f).

**Figure 4:**
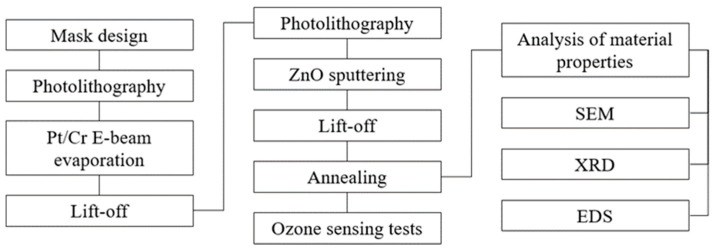
Flowchart showing main steps in proposed research system.

As is shown in Figure 4, the main components of this study include mask design, process development, material analysis, and sensing performance evaluation. For both ZnO sensors (the SAW and resistive types), platinum is selected as the electrode material, with a thin layer of chromium used as an adhesion layer between the electrode and quartz glass substrate. In the fabrication process, standard photolithography techniques are used to transfer the mask pattern to the substrate, and the electrodes are formed using Pt/Cr electron-beam evaporation followed by metal lift-off. The ZnO sensing layer is sputtered onto the electrodes, and the sensors are then annealed to improve the crystallinity and sensing performance of the ZnO layer. The structure and composition of the sensing layer are characterized using scanning electron microscopy (SEM), X-ray diffraction (XRD), and energy-dispersive spectroscopy (EDS). Finally, sensing tests are conducted under various ozone concentrations and working temperatures.

## 3. Methods and Fabrication

**Figure 5:**
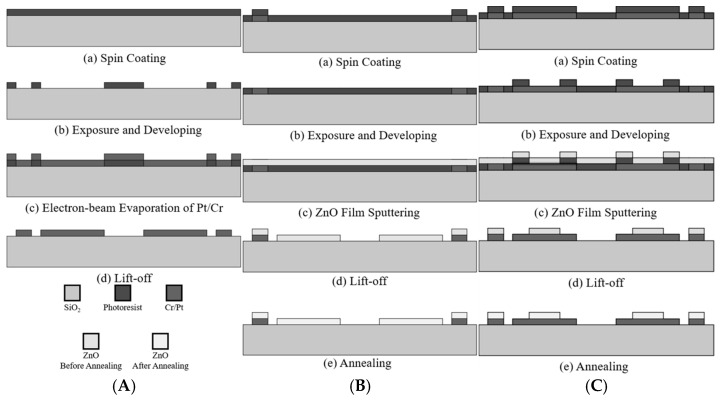
(**A**) Workflow for initial fabrication stage, (**B**) workflow for the second fabrication stage of SAW-type sensor, and (**C**) workflow for the second fabrication stage of resistive-type sensor.

Figure 5A illustrates the main steps in the initial fabrication process for the resistive-type ozone sensor. As is shown, the process commences by spin coating a thin layer of photoresist (PR) onto the substrate surface. The PR layer was selectively exposed and developed to create the required pattern, and electron-beam evaporation was then performed to deposit a thin platinum/chromium metal layer. Finally, a lift-off process was used to remove the excess PR and leave behind only the desired metal electrode pattern on the substrate surface.

Figure 5B shows the workflow for the second stage of the SAW-type-sensor fabrication process. First, a thin layer of PR was evenly applied to the patterned substrate via spin coating. Exposure and development procedures were then performed to define the desired pattern. Next, a ZnO thin film with a uniform thickness was deposited to the substrate through RF sputtering. A lift-off process was then carried out to remove the excess PR and unwanted ZnO layer, leaving behind only the patterned ZnO sensing layer. Finally, the substrate was annealed to improve the crystallinity of the ZnO film and enhance its sensing performance.

Figure 5C shows the main steps in the fabrication process for the resistive elements of the resistive-type ozone sensor. A spin-coating operation was first performed to deposit a thin PR layer on the patterned substrate. Following an exposure and development process, a thin ZnO film was deposited via RF sputtering. The unwanted ZnO was then removed using a lift-off process to leave only the desired patterned ZnO structures on the substrate. The patterned structures were then annealed to enhance their material properties and improve their sensing performance.

**Figure 6:**
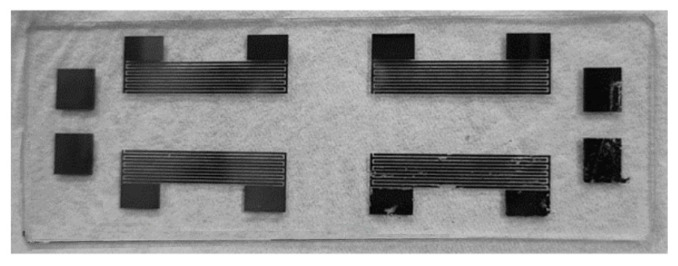
Photograph of electron-beam-evaporated substrate.

**Figure 7:**
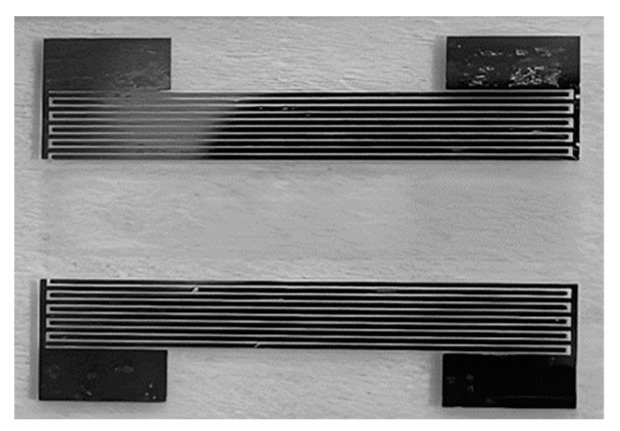
Photograph of annealed substrate.

As is shown in Figure 6, two ozone SAW chips were fabricated on a single quartz glass. Four square alignment marks located on both sides of the photomask were used to ensure the precise alignment of the mask during the patterning process. Figure 7 presents a photograph of the final sensor.

## 4. Results and Discussion

The crystalline structures of the ZnO thin films were analyzed using an X-ray diffractometer (D/max-220/PC, Rikagu, Tokyo, Japan), with a copper target (monochromatic Cu-Kα; λ = 0.154 nm) as the X-ray source. The XRD patterns were obtained using an operating voltage and current of 40 kV and 40 mA, respectively, and a 2*θ* scanning range of 20° to 60°. The sampling interval was set to 0.03° with a sampling time of 0.45 s. The crystalline phases and peak intensities were identified using JCPDS (Joint Committee on Powder Diffraction Standards) cards.

**Figure 8:**
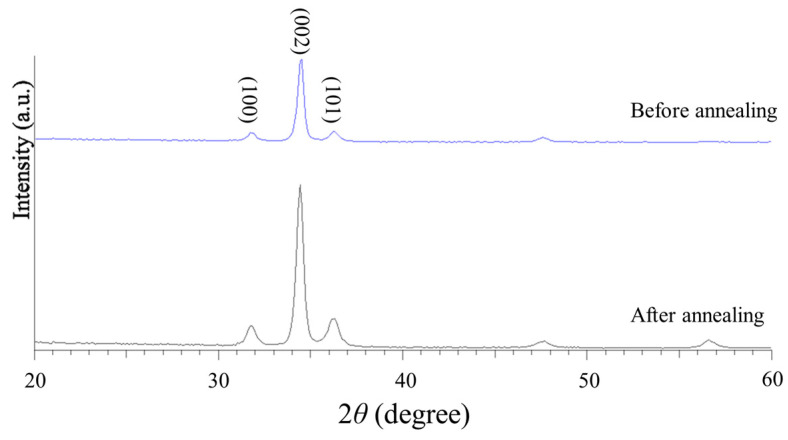
XRD pattern of as-sputtered and annealed ZnO thin films at 500 °C for 4 h.

Figure 8 shows the XRD pattern of the as-sputtered and annealed ZnO thin films at 500 °C for 4 h. A prominent characteristic peak is observed in the (002) crystallographic direction, with a higher diffraction intensity and a narrower full width at half maximum (FWHM). The XRD pattern confirms the effectiveness of the annealing process in improving the crystallinity and structural quality of the ZnO sensing layer.

**Figure 9:**
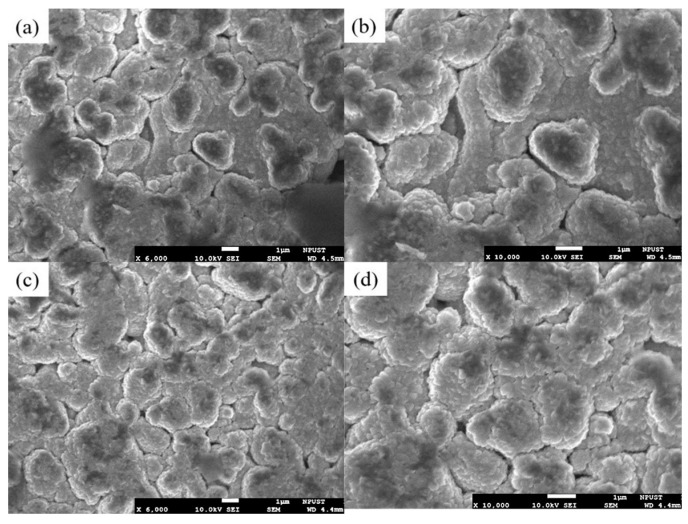
SEM images of as-sputtered and annealed ZnO thin films: (**a**) as-sputtered film (×6000), (**b**) as-sputtered film (×10,000), (**c**) annealed film (×6000), and (**d**) annealed film (×10,000).

The microstructures of the as-sputtered and annealed ZnO films were analyzed using a high-resolution field-emission scanning electron microscope (FE-SEM, JSM-7600F, JEOL, Tokyo, Japan). As is shown in Figure 9a,b, the as-sputtered ZnO film had a loose microstructure with no apparent crystalline formation. In contrast, the film that was annealed at 500 °C for 4 h exhibited significant crystalline growth and the formation of voids at the grain boundaries due to the thermal stress induced during the annealing process (Figure 9c,d). The voids increase the contact area between the sensing layer and the target gas, therefore enhancing the gas sensitivity. The annealing process also expands the depletion layer, leading to more substantial resistance changes and a greater sensor sensitivity. Overall, the results demonstrate that annealing at 500 °C not only improves the crystallinity of the ZnO film but also significantly enhances its sensing performance.

**Figure 10:**
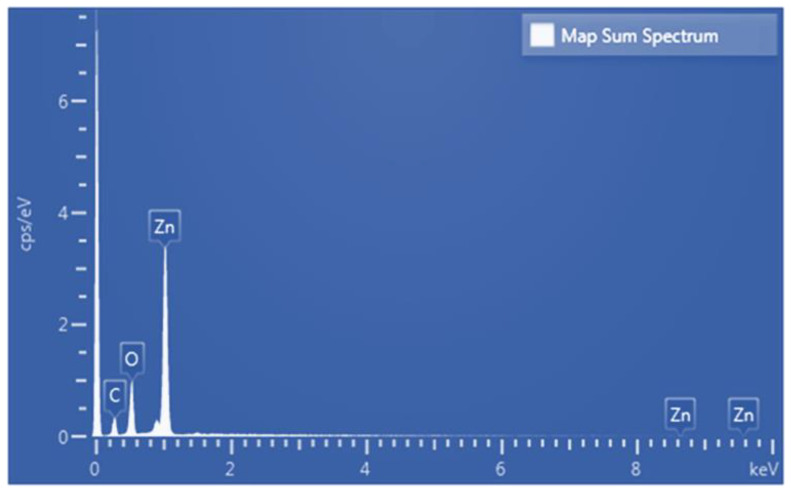
EDS spectrum of annealed ZnO thin film.

Figure 10 present the EDS analysis results for the annealed ZnO thin film. The results show that the film has a Zn content of 30.89 at% and an O content of 29.65 at%. The Zn-to-O atomic ratio is thus close to 1:1, which is consistent with its stoichiometric ratio. The carbon content (39.47 at%) in the annealed film originates from the conductive carbon tape used during sample preparation and indicates that the sputtered film contains no extraneous impurities. The weight percentages also correspond to the atomic weight ratio of ZnO (Zn: ~65.38; O: ~16), further verifying the purity of the sputtered film.

**Figure 11:**
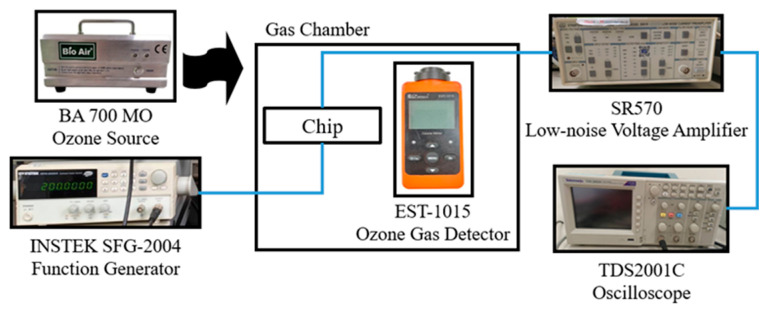
Experimental setup for SAW-type sensor characterization.

The performance of the SAW- and resistive-type sensors was evaluated under ozone concentrations ranging from 0 to 5 ppm. For reference purposes, the ozone concentration was also measured using a commercial electrochemical ozone detector (EST-1015, EST, Miami, FL, USA). In the test procedure, ozone gas was supplied via a BA 700 MO ozone generator (Bio Air, Taipei, Taiwan). For the SAW-type sensor, the input signal was provided by an INSTEK SFG-2004 function generator (Instek Digital, New Taipei, Taiwan), while the output signal was passed through an SR570 low-noise voltage amplifier (TEO, Taipei, Taiwan) and displayed on a TDS2001C oscilloscope (Tektronix, Beaverton, OR, USA). The experimental arrangement of the SAW-type sensor is shown in Figure 11.

**Figure 12:**
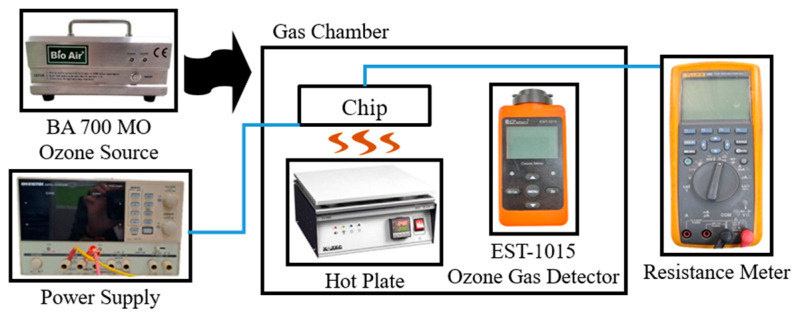
Experimental setup for resistive-type ozone sensor characterization.

The resistive-type ZnO sensor was mounted on a hot plate to maintain the specified operating temperature, and ozone was introduced into the chamber via the BA 700 MO generator (Figure 12). The corresponding change in the chip resistance was measured using a resistance meter. Reference values of the ozone concentration were again obtained using the EST-1015 commercial electrochemical ozone detector (EST, Miami, FL, USA).

**Figure 13:**
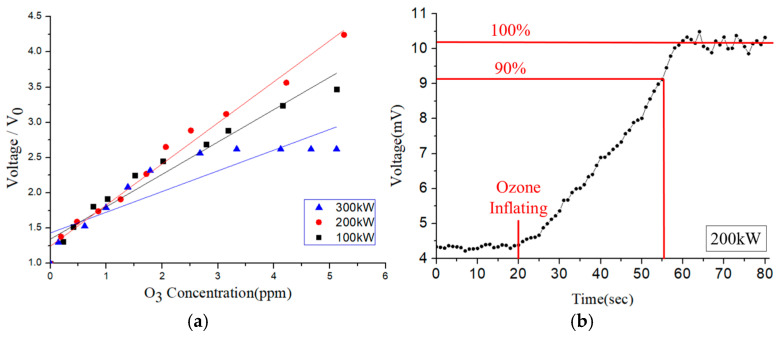
(**a**) Sensitivity of SAW-type ozone sensor and (**b**) response time of SAW-type ozone sensor.

Figure 13a shows the variation in the voltage signal of the SAW-type sensor with the ozone concentration for three different settings of the function-generator power (100 kW, 200 kW, and 300 kW). For all three settings, the voltage increases approximately linearly with increasing ozone concentration. The response time is defined as the time to reach a 90% change during gas adsorption [22]. Among the three settings, the sensor response that was achieved using a power of 200 kW showed the best linearity and greatest slope (i.e., the best sensitivity). Thus, it was chosen as the optimal power setting for subsequent studies. Figure 13b shows the response time of the sensor in the presence of ozone with a concentration of 2.5 ppm. It is seen that the 90% response time is approximately 35 s.

**Figure 14:**
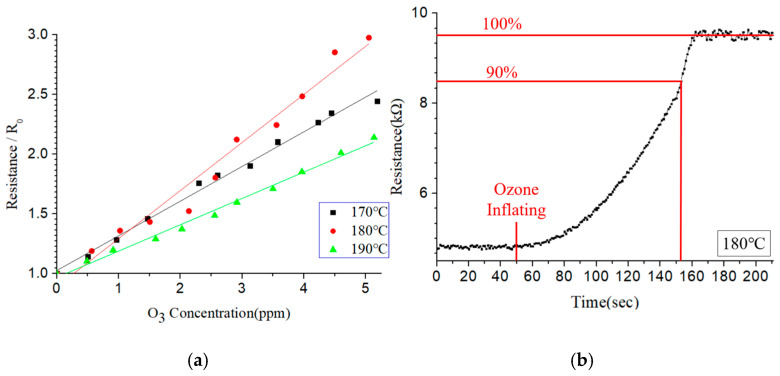
(**a**) Sensitivity of resistive-type ozone sensor and (**b**) response time of resistive-type ozone sensor.

Figure 14a shows the output response of the resistive-type sensor under ozone concentrations of 0–5 ppm for three different operating temperatures (170 °C, 180 °C, and 190 °C). The sensitivity is defined as the resistance change divided by the ozone-concentration change [23]. The sensor shows a linear response at all three temperatures. However, the optimal sensitivity (i.e., the greatest slope) is observed at 180 °C. Figure 14b shows the response time of the sensor under an ozone concentration of 2.5 ppm. The 90% response time is around 103 s.

**Figure 15:**
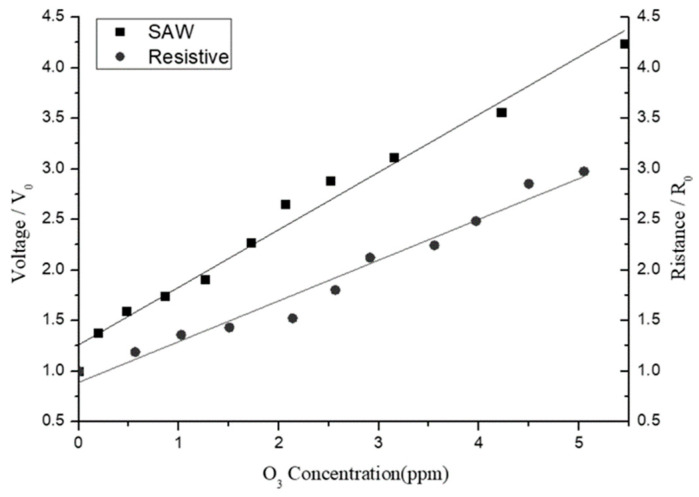
Sensitivity comparison of SAW- and resistive-type sensors.

Figure 15 compares the sensitivity responses of the SAW- and resistive-type sensors under ozone concentrations ranging from 0 to 5 ppm. The left y-axis indicates the output voltage ratio (V/V_0_) of the SAW-type sensor, while the right y-axis represents the resistance ratio (R/R_0_) of the resistive-type sensor. Both axes are normalized with respect to their initial values (no ozone) to obtain comparable units for analysis purposes. For both sensors, the output signal increases approximately linearly with an increasing ozone concentration. However, the SAW-type sensor shows an improved linearity and a greater voltage variation. Thus, over the considered low-ozone-concentration range (0–5 ppm), it provides a better sensing performance than the resistive-type sensor.

**Figure 16:**
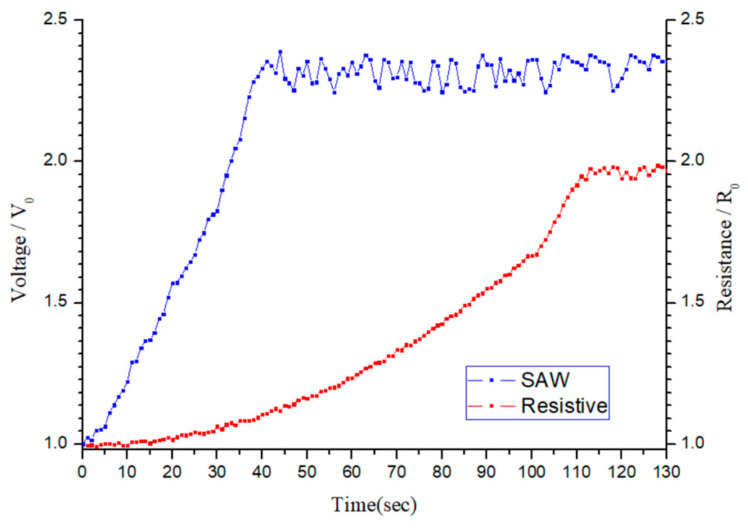
Response-time comparison of SAW- and resistive-type sensors.

Figure 16 compares the time response characteristics of the SAW- and resistive-type sensors. For both sensors, the normalized output signal increases progressively over time and eventually stabilizes. However, the SAW-type sensor exhibits a faster response, reaching a stable value within approximately 35 s, demonstrating a greater change in the output voltage. In contrast, the resistive-type sensor requires around 103 s to achieve a stable response and exhibits a smaller change in the output signal. Furthermore, the resistive sensor requires an operating temperature of 180 °C to optimize the sensing performance. In other words, compared to the SAW-type sensor, the resistive-type sensor not only has an inferior sensitivity but also has a higher energy consumption and hence a greater operating cost.

Overall, the results suggest that the SAW-type sensor provides a more feasible solution for real-time ozone-monitoring applications. However, despite its slower response and heating requirements, the resistive-type sensor offers good stability and a simpler manufacturing process. Consequently, it provides an attractive solution for long-term stable ozone monitoring and large-scale deployment.

## 5. Conclusions

This study has successfully fabricated surface acoustic wave (SAW)- and resistive-type ozone sensors based on zinc oxide (ZnO) thin films. For ozone concentrations in the range of 0–5 ppm, the SAW-type sensor exhibited the highest sensitivity at a frequency of 200 kHz, with a response time of only 35 s. Thus, due to its dedicated IDT design and working principle, it provides a feasible solution for ozone sensing applications requiring a rapid response time and high precision, such as real-time environmental monitoring and precision instrumentation. The resistive-type sensor exhibited an optimal sensitivity at an operating temperature of 180 °C but had a relatively long response time of 103 s. Consequently, compared to the SAW-type sensor, it provides a more suitable solution for low-cost and large-scale industrial applications, such as gas-concentration monitoring in industrial settings. Overall, the findings validate the potential of ZnO materials for ozone sensing and provide a useful technical reference for future sensor designs.

## Data Availability

The data presented in this study are available on request from the corresponding author.
